# *Metabolites* Best Paper Awards for 2015

**DOI:** 10.3390/metabo5020386

**Published:** 2015-06-17

**Authors:** Peter Meikle

**Affiliations:** Metabolomics Laboratory, Baker IDI Heart and Diabetes Institute, Melbourne, Victoria 3004, Australia; E-Mail: peter.meikle@bakeridi.edu.au

*Metabolites* is instituting annual awards to recognize the most outstanding papers in metabolism and metabolomics published in *Metabolites*.

We are pleased to announce the recipients of the first “*Metabolites* Best Paper Awards”. Nominations were selected by the Editor-in-Chief and several Editorial Board members of *Metabolites* from all original research articles published in 2013 or 2014.

We are pleased to announce that the following three papers were chosen to receive “*Metabolites* Best Paper Awards” for 2015. I congratulate all of the authors and thank them for their significant and important contributions to the field of metabolism and metabolomics. In recognition of their accomplishment, Dr. Larissa Stanberry, Dr. Royston Goodacre and Dr. Anne-Christin Hauschild will receive the privilege of publishing a paper free of charge in open access format in *Metabolites*, respectively, after the usual peer-review procedure. I also thank the members of the Selection Committee for their efforts.

**Article Award**
  First Prize
    **Larissa Stanberry, George I. Mias, Winston Haynes, Roger Higdon, Michael Snyder and Eugene Kolker**
    Integrative Analysis of Longitudinal Metabolomics Data from a Personal Multi-Omics Profile
    *Metabolites* **2013**, 3(3), 741-760; doi:10.3390/metabo3030741
    Available online: http://www.mdpi.com/2218-1989/3/3/741/htm
  Second Prize
    **Piotr S. Gromski, Yun Xu, Helen L. Kotze, Elon Correa, David I. Ellis, Emily Grace Armitage, Michael L. Turner and Royston Goodacre**
    Influence of Missing Values Substitutes on Multivariate Analysis of Metabolomics Data
    *Metabolites* **2014**, *4*(2), 433-452; doi:10.3390/metabo4020433
    Available online: http://www.mdpi.com/2218-1989/4/2/433/htm
  Third Prize
    **Anne-Christin Hauschild, Dominik Kopczynski, Marianna D’Addario, Jörg Ingo Baumbach, Sven Rahmann and Jan Baumbach**
    Peak Detection Method Evaluation for Ion Mobility Spectrometry by Using Machine Learning Approaches
    *Metabolites* **2013**, *3*(2), 277-293; doi:10.3390/metabo3020277
    Available online: http://www.mdpi.com/2218-1989/3/2/277/htm
***Award Selection Committee***
  Editor-in-Chief
    **Dr. Peter Meikle**
    Metabolomics Laboratory, Baker IDI Heart and Diabetes Institute, Melbourne, Victoria 3004, Australia
    E-Mail: peter.meikle@bakeridi.edu.au
  Editorial Board Member
    **Dr. Jan Baumbach**
    Computational Biology Department, Institute for Mathematics and Computer Science, University of Southern Denmark, Campusvej 55, 5230 Odense C, Denmark
    E-Mail: jan.baumbach@imada.sdu.dk
  Editorial Board Member
    **Dr. Aalim Weljie**
    Department of Pharmacology, University of Pennsylvania, 10-113 Translational Research Center, 3400 Civic Center Blvd, Bldg 421, Philadelphia, PA 19104 USA
    E-Mail: aalim@mail.med.upenn.edu
  Editorial Board Member
    **Prof. Dr. Pollen K.F. Yeung**
    Pharmacokinetics and Metabolism Laboratory, 5968 College Street, Burbidge Building, Dalhousie University, Halifax, Nova Scotia, B3H 4R2, Canada
    E-Mail: Pollen.Yeung@Dal.Ca
  Editorial Board Member
    **Dr. Wolfgang Eisenreich**
    Technische Universität München, Department Chemie, Lichtenbergstraße 4, 85747 Garching, Germany
    E-Mail: wolfgang.eisenreich@mytum.de
  Editorial Board Member
    **Prof. Dr. Monica Colitti**
    Department of Agricultural and Environmental Sciences (DISA) via delle Scienze, 206 - 33100 Udine Italy
    E-Mail: monica.colitti@uniud.it

